# Plant cell (*Brassica napus*) response to europium(III) and uranium(VI) exposure

**DOI:** 10.1007/s11356-020-09525-2

**Published:** 2020-06-06

**Authors:** Henry Moll, Susanne Sachs, Gerhard Geipel

**Affiliations:** Helmholtz-Zentrum Dresden-Rossendorf, Institute of Resource Ecology, Bautzner Landstrasse 400, 01328 Dresden, Germany

**Keywords:** Uranium, Europium, Plant cells, Luminescence spectroscopy, Viability

## Abstract

Experiments conducted over a period of 6 weeks using *Brassica napus* callus cells grown *in vitro* under Eu(III) or U(VI) stress showed that *B. napus* cells were able to bioassociate both potentially toxic metals (PTM), 628 nmol Eu/g_fresh cells_ and 995 nmol U/g_fresh cells_. Most of the Eu(III) and U(VI) was found to be enriched in the cell wall fraction. Under high metal stress (200 μM), cells responded with reduced cell viability and growth. Subsequent speciation analyses using both metals as luminescence probes confirmed that *B. napus* callus cells provided multiple-binding environments for Eu(III) and U(VI). Moreover, two different inner-sphere Eu^3+^ species could be distinguished. For U(VI), a dominant binding by organic and/or inorganic phosphate groups of the plant biomass can be concluded.

**Electronic supplementary material:**

The online version of this article (10.1007/s11356-020-09525-2) contains supplementary material, which is available to authorized users.

## Introduction

The transfer of radionuclides such as actinides through the environment represents a critical safety concern for both nuclear waste repositories and former uranium mining and milling sites that must be made secure. Similarly, the potential harm associated with the growing use of lanthanides, for instance in high-tech products, has resulted in an elevated release of these elements into the environment, which may also represent severe health risk for humans. Due to the fact that lanthanides and actinides display many similarities based on their comparable ionic radii for elements of the same oxidation state and their analogous aqueous chemistry, lanthanides are considered to be suitable chemical analogs for actinides from a (bio-)chemical point of view. For instance, Eu(III) represents an analog for the trivalent actinides americium(III) and curium(III).

The accumulation of radionuclides and other potentially toxic metals (PTMs) into plants, and thus into the food chain, represents a potential pathway for human exposure. Plants need trace elements, e.g., copper and zinc, which represent important micronutrients for metabolic maintenance. However, in higher concentrations, all metal ions are toxic. Actinides and lanthanides, e.g., uranium and europium, are generally non-essential elements and are unlikely to have a special route for transport into plants; nonetheless, they can be taken up by plants and may interfere with normal metabolic processes. For instance, PTMs can replace essential metal ions from their binding sites in enzymes, damage sulfhydryl-group-containing proteins, accelerate the formation of reactive oxygen species, and trigger antioxidant defense mechanisms in plants (e.g., Weiler and Nover 2008; Serre et al. 2019; Aranjuelo et al. 2014). To overcome this unwanted chain of events, plants synthesize protective metal binding metabolites, store metal chelates in vacuoles or secrete them into the rhizosphere (Weiler and Nover 2008), and deposit defense polymers such as callose or lignin (Serre et al. 2019).

The interaction of actinides and lanthanides with plants is often described in terms of transfer factors. In contrast, studies exploring the underlying mechanisms of these interactions at the cellular level, or those investigating toxic metal speciation at the molecular level, are less common. A recent study described the physiological and cellular responses of *Arabidopsis thaliana* roots to U stress (Serre et al. 2019); among several findings, the authors reported the deposition of the defense polymers callose and lignin in the roots due to uranium stress. Earlier studies of the interaction of uranium with plants revealed, for example, the importance of radionuclide speciation for the uptake and translocation of radionuclides in plants (e.g., Ebbs et al. 1998; Laurette et al. 2012a, 2012b), as well as the effects of uranium on phosphate homeostasis regulation (Misson et al. 2009; Berthet et al. 2018). In addition to the speciation effects on uranium uptake and the oxidative stress response (Saenen et al. 2013, 2015), the redox state of uranium and the influence of uranium on the intracellular glutathione pool of plants have also been investigated (Viehweger et al. 2011). The in situ speciation of uranium in plants (Günther et al. 2003) and their subcellular compartments (Geipel and Viehweger 2015) have been confirmed by spectroscopy. In a recent study, Sachs et al. (2017) combined isothermal microcalorimetry with spectroscopy and thermodynamic modeling to investigate the correlation between U(VI) toxicity in plant cells with oxidoreductase activity and U(VI) speciation. Earlier, Drake et al. (1997) used lanthanide ion probe spectroscopy in order to characterize the Eu^3+^ binding sites on *Datura innoxia* cell wall fragments. Similarly, Eu^3+^ uptake and partitioning on the common oat (*Avena sativa*) were investigated using time-resolved laser-induced fluorescence spectroscopy (TRLFS) and confocal microscopy profiling (Fellows et al. 2003). The authors confirmed the existence of Eu^3+^ inner-sphere mononuclear complexes within the root. The impact of the europium speciation on its accumulation in *Brassica napus* and over-expressing *BnTR1* lines was studied by Zha et al. (2014).

The utilization of in vitro callus cell cultures represents an effective method for studying the physiological and biochemical response mechanisms to several stress factors at the cellular level (e.g., Huang et al. 2017a). Principally, callus cells are superior to the intact plant due to the simpler organization of their cells and tissues, thus augmenting the ability to more tightly control their growth conditions. Moreover, as discussed by Zagoskina et al. (2007), this approach also facilitates the ability to synthesize secondary metabolites that are characteristic of intact tissues. Callus cells have already been used to study the impact of PTMs on the growth of plant cell tissues. Maróti and Bognár (1989) investigated the growth inhibition of *Ruta graveolens* L. callus tissues in the presence of varying amounts of Cd, Cu, Hg, Ni, Pb, and Zn. Some years later, the effects of Cu on callus growth and the gene-expression of explants of *Nicotiana glauca* were reported by Taddei et al. (2007). The impact of Cu stress on the growth of castor bean callus cells was studied in vitro by Huang et al. (2017a), who were able to determine the distribution and the chemical form of Cu in the cells. Conversely, there is currently a lack of knowledge on the interaction of callus cell cultures (*Brassica napus*) with actinides and lanthanides with regard to their bioassociation and distribution, as well as their impact on cell growth and metabolism. Moreover, the speciation of actinides and lanthanides in callus cells and their cell compartments has yet to be fully investigated.

Accordingly, this study was designed to determine the tolerance of canola (*Brassica napus)* callus cells to U(VI) and Eu(III) at two different metal concentrations. The effects of both PTMs on cell growth and vitality, as well as on the total phenolic content of the cells, were studied. Furthermore, this investigation also focused on the speciation of bioassociated U(VI) and Eu(III) and their distribution in various fractions of *B. napus* cells, since *B. napus* is known to be able to accumulate PTMs in higher quantities than many other species (Laurette et al. 2012b).

## Materials and methods

### Cell cultivation in the presence of Eu(III) and U(VI)

*Brassica napus* callus cells were obtained from DSMZ (PC-1113, Braunschweig, Germany). The cells were cultivated in a 4-week growth cycle in the dark at room temperature on a solid modified Linsmaier and Skoog medium (medium R) containing 0.8% agar (Linsmaier and Skoog 1965). The callus cells were grown on a solid medium R with a reduced phosphate concentration of 6.25 × 10^−6^ M (medium R_red_, Tab. SI1) supplemented with 20 or 200 μM UO_2_(NO_3_)_2_ or 30 or 200 μM EuCl_3_ (99.999%, Aldrich, Taufkirchen, Germany). The original phosphate concentration of the medium was reduced to minimize the precipitation of Eu(III) and U(VI) phosphate complexes.

Friable callus cells (400 mg) were transferred into Petri dishes (Roth, Karlsruhe, Germany) with the respective PTM-containing medium R_red_. The Petri dishes were then sealed with Parafilm®M (Bemis, Braine L’Alleud, Belgium) and stored in the dark at room temperature. Control samples lacking either Eu(III) and U(VI) were prepared under the same conditions. Eight independent experiments were performed with at least three, and at most ten, parallel samples used for controls with each heavy metal concentration. Cell growth was monitored every week. After about 6 weeks, the cells were collected from the solid medium, combined, and the resulting total weight was determined. In order to study the metal bioassociation by the cells as a function of the exposure time, samples from four experiments were collected weekly and analyzed.

### Determination of the Eu(III) and U(VI) bioassociation by the cells

In order to determine the amount of bioassociated Eu(III) and U(VI), which represents the sum of the metal sorbed onto the cells and taken up by the cells, about 100 mg fresh cells were weighed into 15-mL Greiner tubes (Greiner, Bio-one, Frickenhausen, Germany). A mixture of 2-mL concentrated HNO_3_ (≥ 65%, p.a., Roth) and 1.5 mL 30% H_2_O_2_ (p.a., stabilized, Roth) was added; the cells were heated in a water bath at 80 °C for 7 h to digest the cells. Then, Milli-Q water was added to the solutions to reach 10 mL total volume, which were then analyzed for their U, Eu, Mg, and Ca content by inductively coupled plasma-mass spectrometry (ICP-MS; model ELAN 9000, PerkinElmer, Boston, MA). The results represent mean values and standard deviations of the mean.

### Vitality measurements

The cell vitality was determined using an MTT assay (Mosmann 1983) as described in Sachs et al. (2017). This approach measures the activity of mitochondrial and cytosolic dehydrogenases, which reduces the yellow, water soluble 3-(4,5-dimethylthiazol-2-yl)-2,5-diphenyl-tetrazolium bromide (MTT) to a blue, water-insoluble formazan product (Lindl and Gstraunthaler 2008).

After cell exposure to Eu(III) or U(VI), 50 mg of fresh cells were weighed into 1.5-mL reaction tubes (Greiner) followed by the addition of 1-mL phosphate-buffered saline solution without Ca^2+^ and Mg^2+^ (PBS; Biochrom, Berlin, Germany) and 200 μL MTT solution (5 mg/mL; Duchefa, Harlem, The Netherlands). Subsequently, the assay was performed as described in Sachs et al. (2017). The vitality of the Eu(III) and U(VI) exposed cells was determined as a percentage of the control samples according to Eq. (1).

1 $$ \mathrm{Cell}\ \mathrm{vitality}\ \left(\%\mathrm{of}\ \mathrm{control}\right)=\frac{\mathrm{Absorbance}\ \mathrm{of}\ \mathrm{exposed}\ \mathrm{cells}}{\mathrm{Absorbance}\ \mathrm{of}\ \mathrm{control}\ \mathrm{cells}}\times 100 $$

The results represent data from five independent experiments, each with two to three samples for control and each metal concentration.

### Estimation of phenolic compounds

The total phenolic content of the callus cells after 39 to 43 days growth time in the absence or presence of Eu(III) or U(VI) was estimated based on Ainsworth and Gillespie (2007). About 100 mg of the fresh cells were added to 1.5-mL reaction tubes (Greiner Bio-one) and then immediately frozen in liquid nitrogen. Subsequently, 1 mL of ice-cold 95% (vol/vol) methanol (Roth) was added to the frozen cells after which the cells were homogenized by applying a plastic pestle. The samples were incubated in the dark for 48 h at room temperature. After incubation, the samples were centrifuged (13,000×*g*, 5 min, room temperature; centrifuge 5415R, Eppendorf, Hamburg, Germany) and the supernatants were separated. A total of 100 μL of each supernatant was pipetted into fresh 1.5-mL reaction tubes; 200 μL of 10% (vol/vol) Folin-Ciocalteu reagent (2 N; Merck, Darmstadt, Germany) was added and the samples were thoroughly mixed on a vortex mixer (Reax control, Heidolph, Schwabach, Germany). After 1–2 min, 800 μL 0.7 M Na_2_CO_3_ (p.a., Roth) solution was added to each tube. All samples were thoroughly mixed and subsequently incubated in the dark for 2 h at room temperature. Under the same conditions, both standard and blank solutions were prepared starting with 0.05–1.0 mM gallic acid (98%, Acros, Geel Belgium) stock solutions in 95% methanol and 95% methanol, respectively. After incubation, 8 × 100 μL sample, standard, or blank solutions were transferred into 96-well plates and the absorbance at 620 nm was measured in a microplate reader (Mithras LB940, Berthold, Bad Wildbad, Germany). A standard curve was calculated from the blank corrected gallic acid standards considering three phenol equivalents per gallic acid molecule. The total phenolics of the blank corrected samples were estimated using the regression equation from the gallic acid standard curve. The results represent mean values and standard deviations of the mean of the eight independent experiments with 25 individual samples for control and each metal concentration.

### Statistical analyses

The statistical evaluation of selected experimental data (cell growth, cell vitality, phenolic compounds, and Ca(II) + Mg(II) cell contents) was performed by the two-tail Student’s *t* test. The statistical analyses were done using the implemented functions in the “Analysis ToolPak” of Microsoft Excel 2010. The *p* value was used to discriminate between data groups showing significance (< 0.05) and those that were not. One asterisk (*p* value less than 0.05) denotes statistical significance and two asterisks is a measure for very significant events (*p* value less than 0.01). *p* values less than 0.5 can be interpreted by a tendency visible in the course of experimental data. The boxplots were prepared also with Microsoft Excel 2010.

### Cell fractionation experiments

Cells that were grown under identical conditions were collected from the solid medium R_red_ and combined to a bulk sample. Approximately, 3 g of the cells was suspended in 5 mL ice-cold 0.154 M NaCl (p.a., Roth) for Eu(III) or 0.154 M NaNO_3_ (99%, Sigma, Steinheim, Germany) for U(VI). The pH of the NaCl and NaNO_3_ solutions was 5.8. These suspensions were then transferred to a glass homogenizer where the cells were homogenized. Cell fractions were separated by differential centrifugation of the cell homogenate (15 min at 1000×*g*; 15 min at 30,000×*g*; 60 min at 50,000×*g*) (Centrifuge 5804R, Eppendorf; Sorvall Evolution RC, Kendro, Langenselbold, Germany). Pellet 1 consisted of heavy cell residues. Pellet 2 represents the lighter cell components (e.g., cell organelles). Pellet 3 contained the membrane-containing fraction and destroyed cell organelles. The last supernatant represents the soluble components (e.g., macromolecular organic matter and inorganic ions including soluble fractions from broken organelles) in the cytosol. The assignment of the fractions was based on the work of Carrier et al. (2003). The U(VI) and Eu(III) speciation in all three pellets and the cytosol fractions were investigated by TRLFS as described below.

In order to determine the Eu(III) and U(VI) content of the individual fractions, aliquots of Pellets 1 to 3 were digested with a mixture of HNO_3_ and H_2_O_2_ as described above. The resulting solutions were analyzed together with the cytosol fraction that was acidified with HNO_3_ for their U and Eu content by ICP-MS.

### Time-resolved laser-induced fluorescence spectroscopy measurements

#### Europium(III)-TRLFS

Eu(III) TRLFS studies were performed as described in Moll et al. (2009) and Moll et al. (2014). Resuspended cells and the cell fractions were measured, which were placed in 1-cm quartz glass cuvettes (Hellma Analytics, Mühlheim, Germany). For this phase of the investigation, cells or cell fractions were suspended in 0.154 M NaCl (pH 5.8) or measured directly at room temperature. Static emission spectra were recorded from 564 to 648 nm with the 1200 lines/mm grating and a resolution of 0.2 ms. For time-resolved measurements (600 lines/mm grating), a dynamic step width was used to describe species with a long emission lifetime, as well as species with short emission lifetime. The following formula was used:

2 $$ {t}_i={t}_0+{F}_1+{F}_2\cdotp x $$

*t*_0_   initial delay, set to 1 μs

*F*_1_   factor 1, set to 5 μs

*F*_2_   factor 2, set to 1 μs

*i*   number of spectrum

*x*   number of previous spectrum

The abatement of the luminescence was investigated over 50 time points, resulting in 50 spectra. The spectra of individual samples were averaged, baseline and energy-corrected, and normalized. The spectra were normalized to the area of the ^7^F_1_ band of Eu(III) with OriginPro 8.6.0G (OriginLab Corporation, USA). Luminescence emission lifetimes were determined with a non-linear fitting as exponential function (ExpDecay1, ExpDecay2) with the same software. The relative peak intensity ratio, R_E/M_, which gives information about the ligand field of Eu(III) and the coordination environment, was determined by forming the ratio for the integral intensities of the ^7^F_2_ to ^7^F_1_ band, as presented in Eq. 3:

3 $$ {R}_{E/M}=\left({}^5{D}_0\to {}^7{F}_2\right)/\left({}^5{D}_0\to {}^7{F}_1\right) $$

The intensities of the transitions (^5^D_0_ → ^7^F_2_) and (^5^D_0_ → ^7^F_1_) were calculated from the corresponding normalized peak areas. The number of coordinated water molecules was determined based on the equations of Kimura and colleagues (Kimura and Choppin 1994; Kimura et al. 1996; Kimura and Kato 1998), which is presented for europium in Eq. 4:

4 $$ {N}_{{\mathrm{H}}_2\mathrm{O}}=1.07{k}_{\mathrm{exp}}-0.62 $$

${N}_{{\mathrm{H}}_2\mathrm{O}}$  coordination number of water molecules

*k*_exp_   reciprocal luminescence emission lifetime (ms)

#### Uranium(VI)-TRLFS

U(VI) TRLFS studies were performed as described in Geipel and Viehweger (2015) and Sachs et al. (2017). Fresh cells or cell fractions were suspended in 0.154 M NaNO_3_ (pH 5.8) and deposited into quartz cuvettes (Hellma Analytics). Spectra were recorded at room temperature using a Peltier-controlled cuvette holder (Flash 300; Quantum Northwest, USA), which was set to 293 K. Depending on the uranium concentration, spectra were measured with 40 to 100 laser pulses per spectrum in the wavelength range between 450.4 and 727.0 nm at a resolution of 0.266 nm. The spectra were evaluated with the OriginPro 2015G software (OriginLab Corporation). To confirm the bioassociation of U(VI) with the cells, reference solutions of 20 or 200 μM U(VI) in 0.154 M NaNO_3_ (pH 5.8) were produced and measured using a coupled Minilite I and Minilite II Nd:YAG laser system (Continuum Electro Optics Inc., Santa Clara, USA) with a repetition rate of 10 Hz. The excitation wavelength was set at 266 nm with pulse energies of about 0.3 mJ. The luminescence emission was focused into a spectrograph (iHR 550, Horiba Jobin Yvon GmbH, Munich, Germany) and detected using an intensified camera system (Horiba Jobin Yvon). Spectra were measured by averaging 100 laser pulses per spectrum in the wavelength range between 372.2 and 671.6 nm at a resolution of 0.461 nm.

## Results and discussions

### Cultivation of *B. napus* cells in the presence of Eu(III) and U(VI): Cell growth, vitality, and bioassociation of both metals as a function of their concentration

**Fig. 1:**
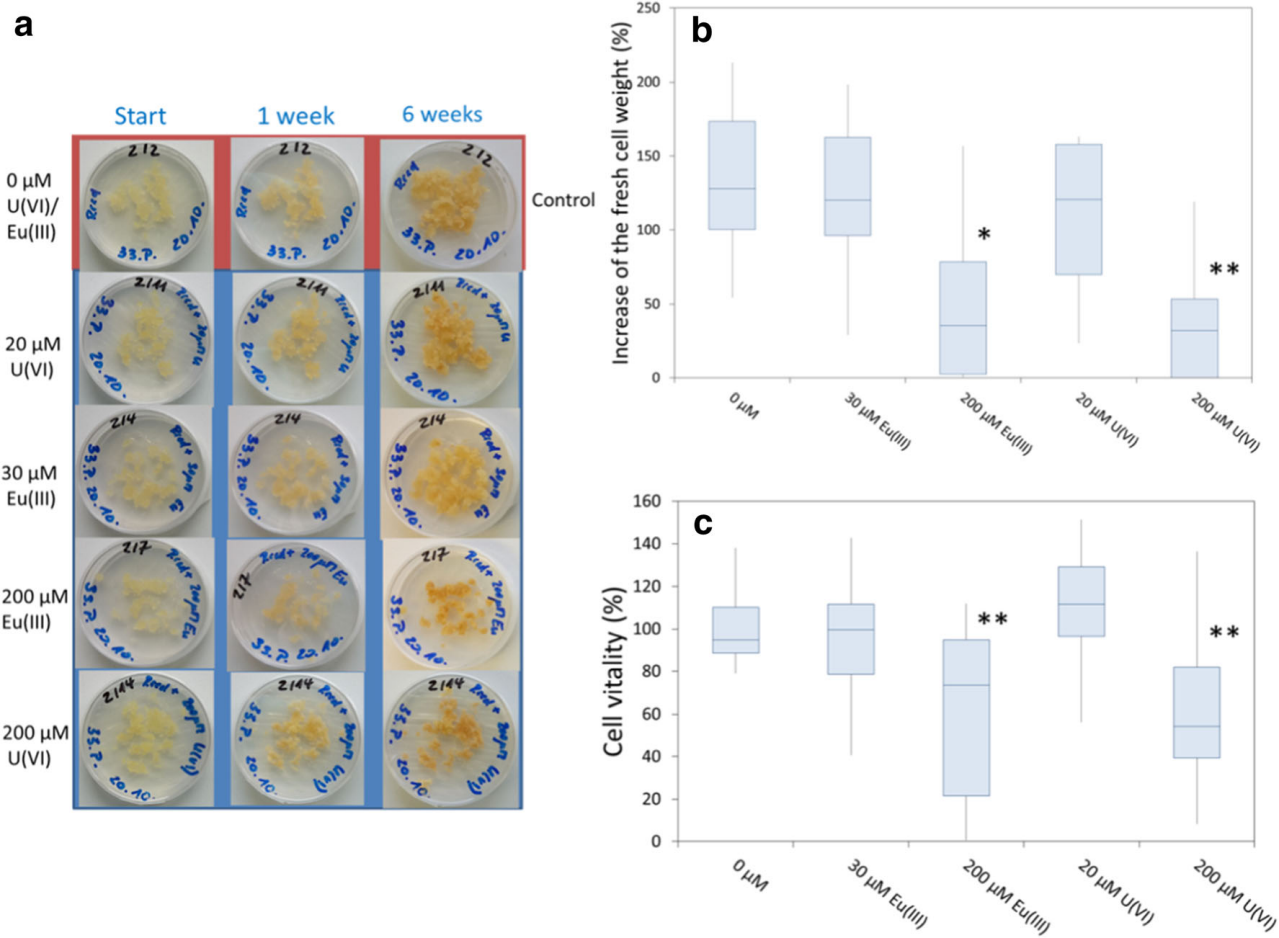
*B. napus* callus cells grown in the presence of different Eu(III)/U(VI) concentrations after different exposure times (a). Box plots for the increase in fresh cell weight (b) and for the cell vitality of *B. napus* callus cells (c) grown on solid medium R_red_ after an exposure time of 6 weeks. Data in Fig. 1b represent measurements from seven independent experiments. For each experiment, accumulated cells from all individual plates were collected and combined in one bulk sample at the end of the experiment. Data in Fig. 1c represent values from five independent experiments, each with 2–3 samples for control and each metal concentration. Significant differences to the untreated cells were calculated by Student’s *t* test and are indicated by *(*p* < 0.05) or **(*p* < 0.01), respectively

Figure 1a illustrates the development of the callus cell samples in the presence of Eu(III) or U(VI) with increasing exposure time compared to control samples. Note that for all samples, both an increase in the amount of cells and a darkening of the cells were visible with increasing growth time.

However, after 6 weeks, the amount of cells grown in the presence of 200 μM Eu(III) or U(VI) appeared to be lower compared to the other samples. This reduction is reflected in the increase of the total cell weight, which is illustrated in Fig. 1b for each sample and the control. For both PTMs, a significantly inhibited cell growth was observed at a concentration of 200 μM, whereas the presence of 30 μM Eu(III) or 20 μM U(VI) did not significantly impact cell growth (Fig. 1b). *B. napus* cells tolerated the low metal concentrations of 20 μM U(VI) and 30 μM Eu(III), with cell vitalities around 100% compared to the control samples; in contrast, a very significant decrease in the cell vitality was measured at 200 μM Eu(III) or U(VI) (Fig. 1c). These data indicate that *B. napus* cells exhibit, to some extent, a resistance against Eu(III) and U(VI). We attribute the inhibited cell growth at 200 μM Eu(III)/U(VI) to the phytotoxic effect of both PTMs due to their high metal concentrations, which is also reflected in lower cell vitality. When we compared cell growth in the presence of Eu(III)/U(VI), we noted slightly higher cell growth for all the Eu(III) assays, which could indicate the positive influence of Eu(III) in trace concentrations on cell metabolism. Notably, a concentration-dependent and element-specific decrease in fresh cell weight has already been reported for *Ruta graveolens* L. callus cells in the presence of Cu, Cd, Zn, Ni, Pb, and Hg (Maróti and Bognár 1989). The cell growth results we observed are also in agreement with the work of Taddei et al. (2007) and Huang et al. (2017a), who reported concentration-dependent callus growth inhibition for *Nicotiana glauca* and *Ricinus communis* L., respectively, in the presence of copper.

**Fig. 2:**
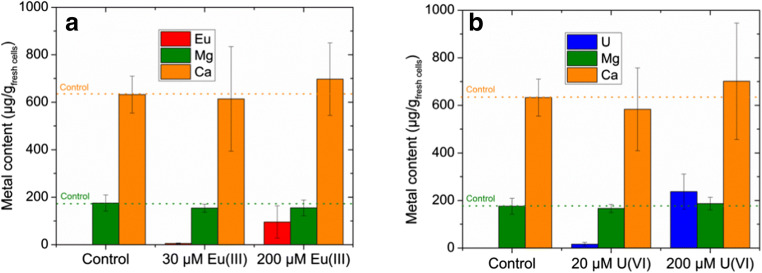
Eu(III) (a) and U(VI) (b) bioassociation by *B. napus* callus cells after an exposure time of 6 weeks and their effects on the homeostasis of intracellular Mg(II) and Ca(II). Data represent mean values ± SD of seven independent experiments for determining the bioassociated amount of Eu(III) and U(VI) after 6 weeks of exposure. The Ca(II) and Mg(II) contents were measured in three independent experiments

The amount of Eu(III) and U(VI) bioassociated to *B. napus* callus cells was determined after 6 weeks exposure time (Fig. 2). In addition, the effects of Eu(III) and U(VI) on the homeostasis of intracellular Mg(II) and Ca(II), the most abundant ions in living systems, were explored and results are illustrated in Fig. 2. At both concentrations, *B. napus* callus cells bioassociated more U(VI), 68 and 995 nmol/g_fresh cells_, than Eu(III), 33 and 628 nmol/g_fresh cells_. Moreover, the intracellular Mg(II) content of the cells had not significantly changed in the presence of Eu(III) and U(VI). The intracellular Ca(II) content appeared to be only slightly higher for cells that were grown in the presence of 200 μM Eu(III) and U(VI). The statistical analysis showed only a slight tendency of the noticed Ca(II) effect. More independent experiments would be needed to verify the postulated Ca(II) effect.

Eu(III) is not only an analogue for trivalent actinides (e.g., Cm(III) and Am(III)), but it serves as an analogue for Mg(II) and Ca(II) as well. To date, research indicates that substituting Eu(III) for Ca(II)/Mg(II) does not appear to impact the normal physiological functions of plant tissues; however, the phytotoxic effects of Eu(III) are unknown (Fellows et al. 2003). Gao et al. (2003) reported that Eu(III) can directly enter into plant cells through the Ca^2+^ ion channel and competes with Ca^2+^ for protein binding sites. U(VI) in the form of the UO_2_^2+^ cation also resembles Ca^2+^ and Mg^2+^ and is able to form complexes with higher stabilities (Vandenhove et al. 2006). Additionally, it is known that U(VI) can replace Ca^2+^ and Mg^2+^, which can lead to structural changes in cell membranes, enzyme inactivation, and damage to RNA and DNA (Saenen et al. 2013). Our observation of a slightly increased Ca(II) uptake with no significant change in the Mg(II) content of the cells in the presence of 200 μM Eu(III) or U(VI) is comparable to the findings of Vanhoudt et al. (2010), who reported enhanced calcium uptake and almost unchanged magnesium concentration in the roots of *Arabidopsis thaliana* seedlings in the presence of uranium and cadmium. Küpper and Kochian (2010) reported that enhanced calcium uptake under cadmium stress in *Thlaspi caerulescens* could impede the replacement of calcium by cadmium in proteins as a defense mechanism against cadmium toxicity. More recently, Huang et al. (2017b) described the protective role of Ca against Cd-induced toxicity in plants. In a similar study, Cao et al. (2018) described how the homeostasis of Ca and Mg in *Camellia sinensis* after Cd treatment was affected at Cd concentrations between 1 and 15 mg/L, noting that the intracellular Ca content in leaves increased with increasing Cd stress, but was much less pronounced for Mg. As documented by Yang et al. (2015), treating horseradish roots with Tb(III) resulted in Tb(III) accumulation in both the extracellular and intracellular spaces of the roots, accompanied by increasing intracellular Ca content as well. These various studies confirm that plant cells do respond to PTM stress with an increased concentration of intracellular Ca.

### Estimation of extractable phenolic compounds

Researchers have documented that plants may synthesize protective metal binding metabolites, e.g., phenolic compounds such as flavonoids or hydrocinnamic acid, in response to PTM stress (Wu et al. 2013). These metabolites can complex the metal ions, store them in the vacuole, or secrete them into the rhizosphere. It should also be noted that phenolic compounds, like flavonoids and lignin precursors, can scavenge harmful oxygen species arising from the unwanted effects of PTM stress (Sytar et al. 2013). In order to study the influence of Eu(III) and U(VI) on the phenolic substance pool of *B. napus* cells, we estimated the total phenolic content of the cells after PTM exposure by applying the Folin-Ciocalteu assay and then comparing results to our control samples. It is important to add that the results of this assay must be interpreted as an estimation, since other oxidizable substrates (i.e., in addition to phenolic compounds) can also react with the Folin-Ciocalteu reagent. In general, an increase in the phenolic content of the cells was detected within the studied time frame. Figure SI1 shows the mean values of the total phenolic content of the metal-exposed plant cells after 6 weeks of exposure to Eu(III) or U(VI) in comparison to analogous data for the control samples. Note that the mean values for the phenolic equivalents exhibited no clear trend based on the different exposure conditions. Moreover, the phenolic content of the cells exposed to 30 or 200 μM Eu(III) turned out to be quite similar to those of the control samples. In the case of U(VI), we detected a slight tendency toward increase in the total phenolic content for those cells exposed to 20 μM U(VI), whereas in the presence of 200 μM U(VI), this value was comparable to those of the controls. Similar behavior was also reported for the effects of Cd exposure on tea plant callus cultures from the roots and stem (Zagoskina et al. 2007). In the presence of 63 μM Cd, an increase in the phenolic content of the cells was observed in comparison to the control sample, whereas in the presence of 106 μM Cd, this value was close to the control.

However, in spite of the darkening of the cells (cf. Fig. 1a) that suggests an increase in the content of lignin and its precursor substances, we did not observe any significant effect of the presence of Eu(III) or U(VI) on the extractable phenolic content of the cells.

### Cell fractionation experiments

**Fig. 3:**
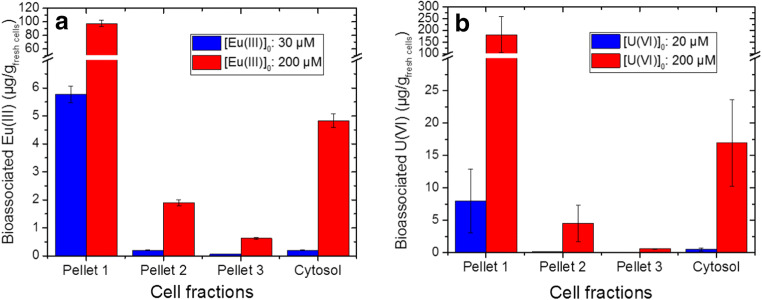
Eu(III) (a) and U(VI) (b) distribution in cell fractions of *B. napus* callus cells after an exposure time of 6 weeks. Data represent mean values ± SD of at least three independent experiments

To determine the speciation of Eu(III) and U(VI) in the different cell compartments of plant cells, cell fractionation experiments were performed. The fractionation protocol described in Geipel and Viehweger (2015) was modified in order to avoid anticipated difficulties in distinguishing the metal speciation in the buffer system and within the cell compartments. Specifically, we replaced the complex buffer solutions with the use of 0.154 M NaCl for Eu(III) and 0.154 M NaNO_3_ for U(VI). The Eu(III)/U(VI) distribution in the different cell fractions is depicted in Fig. 3. As shown therein, most of the Eu(III) and U(VI) was bound onto heavy cell components, e.g., the cell wall fraction (Pellet 1), which can be explained by the function of the cell wall—and its high metal-absorption capacity—as the first barrier in preventing metals from entering the cellular environment. This finding is in agreement with the distribution of copper in castor bean callus cultures (Huang et al. 2017a). It should be noted, however, that a small amount of U(VI) (16 μg/g_fresh cells_ at 200 μM U(VI)) and Eu(III) (5 μg/g_fresh cells_ at 200 μM Eu(III)) was found within the cells in the cytosol fraction, thus indicating that PTM uptake into the cells had occurred (cf. Fig. 3).

### Spectroscopic analysis of Eu(III) and U(VI) speciation

TRLFS is a non-invasive, selective, and highly sensitive method for detecting Eu(III) and U(VI) in the nM to μM concentration range, with resulting spectroscopic data used to conduct speciation analyses in order to determine the local environment of both metals (Geipel 2006; Binnemans 2015).

### Results of Eu(III)-TRLFS

**Fig. 4:**
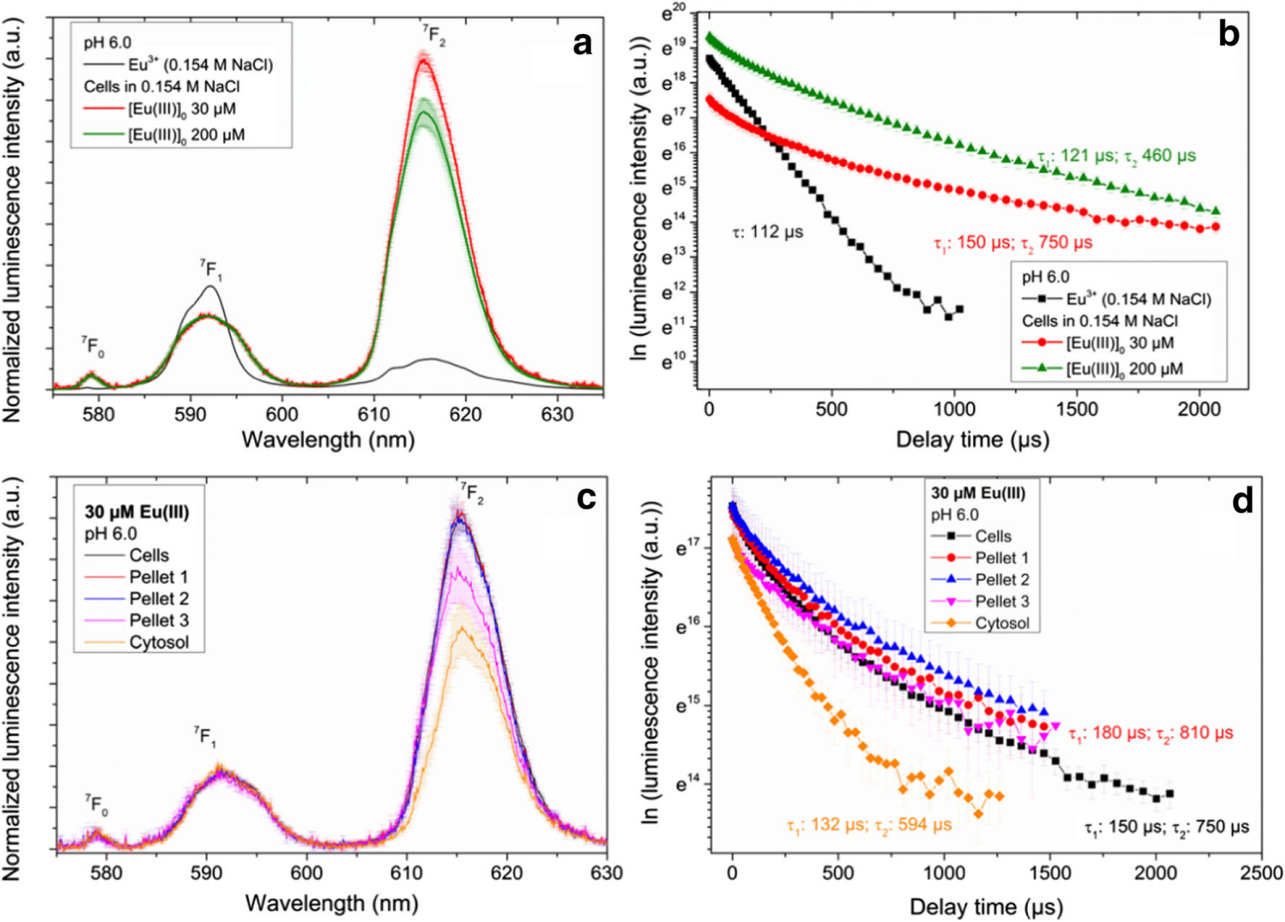
Average luminescence emission spectra and time-dependence of the luminescence decay of Eu(III) in the *B. napus* callus cell system after 6 weeks of incubation. Luminescence spectra of Eu(III)-loaded *B. napus* cells resuspended in 0.154 M NaCl at 30 and 200 μM [Eu(III)]_0_ (a). Corresponding luminescence decay behavior (b). Luminescence spectra of Eu(III) in different cell fractions at [Eu(III)]_0_ 30 μM (c). Corresponding luminescence decay behavior (lifetimes for Pellets 1 to 3 are similar and given in red) (d)

Following an exposure time of 6 weeks, we obtained luminescence spectra of Eu(III) bioassociated by *B. napus* cells as a function of the initial Eu(III) concentration, which are depicted in Fig. 4. Eu(III)-loaded plant cells were either measured directly or suspended in 0.154 M NaCl, with no differences in the luminescence spectra and lifetimes observed. Our results confirmed that the intensity of the hypersensitive ^5^D_0_ → ^7^F_2_ transition at about 616 nm increased strongly; the symmetry­forbidden ^5^D_0_ → ^7^F_0_ transition appeared at around 579 nm (Fig. 4a); and the luminescence decay changed to bi-exponential with prolonged lifetimes (Table 1, Fig. 4b). The luminescence spectrum of the Eu^3+^ aqua ion was shown to be characterized by emission bands at 585–600 nm (magnetic dipole transition ^5^D_0_ → ^7^F_1_) and 610–625 nm (hypersensitive transition ^5^D_0_ → ^7^F_2_). The intensity ratio according to Eq. (3) of 0.5 and the measured lifetime of 112 ± 5 μs corresponding to 9 water molecules in its first coordination sphere are in good agreement with the literature (e.g., Horrocks and Sudnick 1979; Kimura and Choppin 1994; Kim et al. 1994; Moulin et al. 1999; Plancque et al. 2003; Heller et al. 2012; Barkleit et al. 2013).

**Table 1:** F_2_/F_1_ ratio of spectra and luminescence lifetimes measured in the Eu(III)-*Brassica napus* cell system.

Sample composition	F_2_/F_1_ ratio (R_E/M_)	Lifetime 1/μs	N_1_ H_2_O	Lifetime 2/μs	N_2_ H_2_O
Eu^3+^ in 0.154 M NaCl pH 5.8 (30, 200 μM)	0.52 ± 0.03	112 ± 5	8.9 ± 0.2		
30 μM Eu(III)					
30 μM Eu(III) in solid medium R_red_	3.0 ± 0.1	133 ± 5 (92%)	7.4 ± 0.3	364 ± 31 (8%)	2.3 ± 0.2
Eu(III) on callus cells	4.3 ± 0.3	150 ± 6 (69%)	6.5 ± 0.3	750 ± 24 (31%)	0.8 ± 0.03
Pellet 1	4.1 ± 0.3	164 ± 9 (61%)	5.9 ± 0.3	783 ± 36 (39%)	0.7 ± 0.03
Pellet 2	4.1 ± 0.2	173 ± 10 (56%)	5.6 ± 0.3	800 ± 35 (44%)	0.7 ± 0.03
Pellet 3	3.7 ± 0.3	212 ± 8 (57%)	4.4 ± 0.2	853 ± 16 (43%)	0.6 ± 0.02
Cytosol	2.8 ± 0.5	132 ± 5 (79%)	7.5 ± 0.3	594 ± 13 (21%)	1.2 ± 0.03
200 μM Eu(III)					
200 μM Eu(III) in solid medium R_red_	2.7 ± 0.1	125 ± 5 (84%)	7.9 ± 0.3	285 ± 16 (16%)	3.1 ± 0.2
Eu(III) on callus cells	3.7 ± 0.6	121 ± 5 (54%)	8.2 ± 0.3	460 ± 8 (46%)	1.7 ± 0.04
Pellet 1	3.1 ± 0.2	123 ± 7 (48%)	8.1 ± 0.5	470 ± 10 (52%)	1.7 ± 0.05
Pellet 2	2.9 ± 0.1	139 ± 7 (46%)	7.1 ± 0.4	516 ± 10 (54%)	1.5 ± 0.03
Pellet 3	2.8 ± 0.5	170 ± 9 (55%)	5.7 ± 0.3	553 ± 16 (45%)	1.3 ± 0.04
Cytosol	2.3 ± 0.02	144 ± 4 (80%)	6.8 ± 0.2	424 ± 26 (20%)	1.9 ± 0.1
Native *Datura innoxia* cell wall fragments pH 5 (Drake et al. 1997)		263 ± 14	3.4 ± 0.2 ^a^	630 ± 19	1.1 ± 0.03 ^a^
Biorex (carboxylate) pH 5 (Drake et al. 1997)		282 ± 5	3.2 ± 0.2 ^a^	620 ± 17	1.1 ± 0.03 ^a^
Eu^3+^- Oat (*Avena sativa*) roots (Fellows et al. 2003)		345	2.8 ± 0.5		

*N*, number of coordinated water molecules

^a^Calculated in this study

Although the ^7^F_1_ peak should not be influenced by complexation, for all our plant cell suspensions, we observed a slight decrease in intensity combined with a broadening of this transition (cf. Fig. 4a). The interaction of Eu(III) with *B. napus* cells was noted to be especially pronounced in the ^7^F_2_ peak. The Eu(III) bioassociated by the plant cells appeared to be characterized by strongly enhanced R_E/M_ values of 4.3 ± 0.3 and 3.7 ± 0.6 for cells grown in the presence of 30 and 200 μM Eu(III), respectively (Table 1).

This finding indicates that the plant cells established a strong ligand field to Eu(III), with the resulting formation of strong species. We also note that bi-exponential luminescence decay was detected in plant cell samples, indicating the occurrence of two different Eu(III) coordination environments. The lifetime of the short-lived component varied between 120 (200 μM Eu(III)) and 150 μs (30 μM Eu(III)). According to Eq. (4), 8 to 6 coordinated water molecules should remain. Assuming that Eu(III) maintains a nine-fold coordination, 1 to 3 binding sites will be filled up by functionalities of the plant cell envelope. However, the lifetime of the long-lived component varied between 460 (200 μM Eu(III)) and 750 μs (200 μM Eu(III)). According to Eq. (4), only 1 to 2 coordinated water molecules should remain, with 7 to 8 binding sites filled up by functionalities of the plant cell. This strong change in the hydration sphere of Eu(III) points to a bioassociation of Eu(III) with *B. napus* cells. Time-dependent luminescence measurements of Eu(III) (30 and 200 μM) in solid medium R_red_ prior to cell contact (see Fig. SI2 and Table 1) showed a different Eu(III) speciation found on the cells. Calculating the Eu(III), as well as the U(VI) speciation in the solid medium R_red_, was challenging due to missing stability constants with the individual medium components (see Table SI1).

The resulting luminescence emission spectra and the corresponding luminescence decays for the Eu(III) found in the different cell fractions are depicted in Fig. 4c and d. In comparing the spectral parameters of Eu(III) taken up by the plant cells with those in the cell fractions, we noted a decrease in R_E/M_ values. Also, we measured prolonged lifetimes in the sequence cells, Pellet 1, Pellet 2, and Pellet 3 independently of the initial Eu(III) concentration (Table 1). The lowest R_E/M_ values of 2.3 and 2.8 (200 μM and 30 μM Eu(III)) were detected within the cytosol of the inner part of the cell. In contrast to the other fractions, here the short-lived component clearly dominated with 80% of total luminescence decay. This finding points to another Eu(III) speciation in the cytosol than just in the outer areas of the cell fragments. Our results indicate that the sum spectra of Eu(III) bioassociated to *B. napus* cells was dominated by the influence of large cell fragments (e.g., Pellets 1 and 2).

**Fig. 5:**
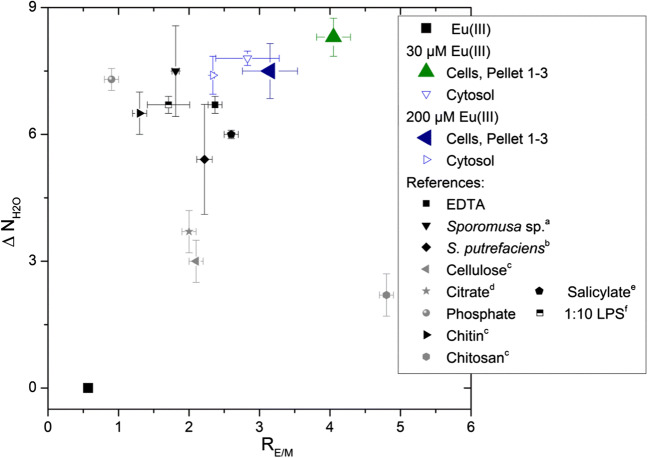
CE diagram of Eu(III) bound to *B. napus* callus cells as Eu(III) species with the long lifetime (y-axis: number of water molecules in the inner-sphere: ΔN_H2O_ = 9-N_H2O_ and x-axis: strength of ligand field R_E/M_ = relative peak intensity ratio according to Eq. (3)). EDTA: ethylenediaminetetraacetic acid, LPS: lipopolysaccharide. References: (a) Moll et al. 2014, (b) Ozaki et al. 2005, (c) Ozaki et al. 2006, (d) Heller et al. 2012, (e) Barkleit et al. 2013, (f) Bader et al. 2019

Based on lifetime measurements, Ozaki et al. (2002) confirmed a relationship between experimentally obtained R_E/M_, the strength of the ligand field, and the geometrical structure around Eu(III). This previously reported empirical approach, which relies on the construction of coordination environment (CE) diagrams, was found to be effective for predicting the coordination environment of both the hydrated and complexed Eu(III) in solutions, as well as that of adsorbed Eu(III) on both the ion-exchange resins and on mammalian cells and microorganisms (Sachs et al. 2015; Moll et al. 2014). Our interpretations of the CE diagrams that we developed are based on the earlier work of Ozaki et al. (2002), as follows. In solutions in which Eu(III) interacts with ligands other than water in an outer-spherical manner, R_E/M_ increases with increasing interaction, whereas ΔN_H2O_ remains small (between 0 and 3), resulting in scattered data from the lower left to the right area of the CE diagram. In the case of predominant inner-sphere coordination, R_E/M_–ΔN_H2O_ data is scattered in the upper-left area of diagram (Fig. 5). Moreover, luminescence measurements as a function of the delay time indicate that the R_E/M_ value of the sum spectrum is influenced principally by the long-lived component. Therefore, the CE diagram presented in Fig. 5 is based on the lifetimes of the long-lived components.

First of all, strong inner-sphere complexes were formed in all samples, which are reflected in the scattered data in the upper part of the diagram (cf. Fig. 5). The Eu(III) coordination environment in the cytosol showed similarities with (a) Eu(III) bound to the bacterial phosphate groups of the cell envelope of *Sporomusa* sp. and bacterial lipopolysaccharide (Moll et al. 2014; Bader et al. 2019), (b) Eu(III) complexed by the strong chelate-ligand EDTA, and (c) Eu(III) complexed by carboxyl groups of salicylic acid (Barkleit et al. 2013). In this first approximation, we confirmed the interaction of Eu(III) with organic phosphate and carboxyl groups in a chelate-manner in the cytosol at the level of [Eu(III)]_0_ 200 μM. Additionally, Eu(III) on/in *B. napus* cells and in the cytosol at the level 30 μM [Eu(III)]_0_ was found to be characterized by an even stronger inner-sphere character.

On closer inspection of the luminescence data of *B. napus* cells exposed to 30 or 200 μM Eu(III), slight differences in the Eu(III) speciation can be deduced. Cells exposed to 30 μM Eu(III) depicted a higher R_E/M_-value and prolonged luminescence lifetimes compared with cells exposed to 200 μM Eu(III). Hence, at 30 μM Eu(III), plant cells established a more intense ligand field to Eu(III). Here both Eu(III) species contained less coordinated water molecules, 1 or 6, respectively. Consequently, more functional groups provided by the cells are involved in the respective Eu(III) species compared with the two Eu(III)-species formed in the presence of 200 μM Eu(III). The more intense Eu(III) interaction to plant cells exposed to 30 μM Eu(III) is also depicted in the CE diagram (cf. Fig. 5).

Drake et al. (1997) assessed ^7^F_0_ → ^5^D_0_ transition excitation spectra to examine the binding sites on native *Datura innoxia* cell wall fragments, with four unique binding sites reported to be involved in metal ion uptake. In particular, the researchers reported that higher-affinity sites tended to involve carboxylates. The native *Datura innoxia* cell wall fragments treated with 300 to 3000 μM Eu(III) at pH 5 also exhibited a bi-exponential luminescence decay with the two lifetimes of 263 and 630 μs (Table 1). The authors concluded that the shorter lifetime is consistent with a 1:1 carboxylate complexation, whereas the longer lifetime indicated that a second and third carboxylate are bound. By comparing these lifetimes with our results, which includes the Biorex values (Drake et al. 1997), an involvement of *B. napus* cell carboxylates seems possible. However, we cannot discount the potential contribution of other functionalities (e.g., phosphate moieties). Most likely, *B. napus* cells provide multiple-binding environments for Eu(III), while some binding sites showed relatively poor luminescent properties. The observed differences, especially for the shorter lifetime, can be attributed to additional processes taking place within living cells in comparison to dead (i.e., inert) biomass. Fellows et al. (2003) conducted an in situ investigation of Eu(III) uptake in oat (*Avena sat*iva) roots by TRLFS, indicating that Eu(III) uptake was highest within undifferentiated root cells. *B. napus* callus cells are also undifferentiated plant cells, which have the propensity to bioassociate a considerable amount of Eu(III). The spectral changes that Fellows et al. (2003) observed when Eu(III) was complexed by cellular components of the oat root indicated the involvement of carboxylic and amino carboxylic functionalities. In summary, the authors linked their data findings to a strong inner-sphere mononuclear Eu(III) complex inside the root with a luminescence lifetime of 345 μs (Table 1). Similarly, our results for Eu(III) in the *B. napus* callus cell system also demonstrated the formation of strong inner-sphere Eu(III) complexes—although the luminescence-decay behavior varied (Table 1). Again, the varying luminescence decay behavior with different lifetime points to multiple-binding environments in living *B. napus* cells.

### Results of U(VI)-TRLFS

**Fig. 6:**
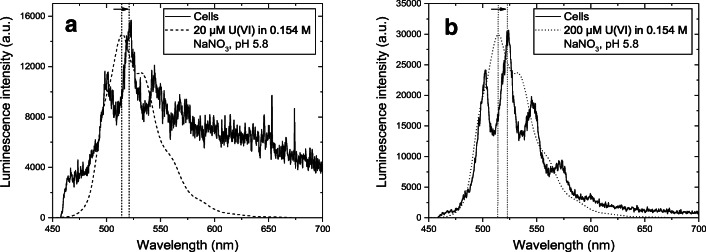
TRLFS spectra of *B. napus* cells cultivated in the presence of 20 μM U(VI) (a: 28 μg U/g_fresh cells_) and 200 μM U(VI) (b: 257 μg U/g_fresh cells_) after 39 days of exposure compared to TRLFS spectra of 20 or 200 μM U(VI) in 0.154 M NaNO_3_ at pH 5.8

Figure 6 provides static luminescence spectra for the *B. napus* cells cultivated in the presence of 20 or 200 μM U(VI) compared to the luminescence spectra of U(VI) in 0.154 M NaNO_3_ (pH 5.8). In contrast to our Eu(III) assays, dynamic quenching processes significantly decreased the luminescence lifetimes of the U(VI) species due to the presence of high amounts of both organic substances and Fe^3+^ and Cl^-^, thereby hampering the ability to compare our findings with model substances as already discussed in Sachs et al. (2017). As a consequence, the measured spectra were not analyzed with regard to their lifetimes. In addition, due to the complex U(VI) luminescence quench processes taking place in the presence of organic substances, TRLFS measurements of the U(VI)-containing solid medium R_red_ were not successful—again, in contrast to our Eu(III) trials. To compare the spectra of the individual samples and to identify dominant U(VI) species, spectra were analyzed by peak deconvolution using the peak-fitting module of OriginPro 2015G and compared to literature data for selected biological systems and U(VI) reference compounds (Table SI2).

Due to the low U(VI) concentration, the spectra of the cells that were cultivated in the presence of 20 μM U(VI) appeared to be dominated by the unspecific self-luminescence of the plant cells. Depending on the amount of bound U(VI), peaks were detected that point to the occurrence of bioassociated U(VI) (Fig. 6a). In contrast, spectra associated with cells grown in the presence of 200 μM U(VI) showed characteristic peaks, clearly indicating the occurrence of bioassociated U(VI) (Fig. 6b). An analysis of the spectra indicates a significant bathochromic shift of the emission bands of the plant cell species when compared to the spectra of the reference solutions of U(VI) in 0.154 M NaNO_3_, which were dominated by the (UO_2_)_3_(OH)_5_^+^ species (cf. Fig. 6, Table SI2). This result points to the biologically induced binding of U(VI)—either extracellular on the cell surface or intracellular. A comparison of the main peak positions with those of U(VI) reference compounds indicates the dominant binding of U(VI) by organic and/or inorganic phosphate groups of the plant biomass (cf. Tab. SI2). However, given the current state of knowledge, we were unable to distinguish between the coordination of the organic and inorganic phosphate groups of the cells. In addition, the dominant binding of U(VI) by carboxyl functionalities does not seem to be very important based on the fact that U(VI) carboxylate reference compounds demonstrated less pronounced bathochromic shifts of their emission peaks (cf. Tab. SI2). Nonetheless, based on the data we obtained, we cannot exclude the potential, but minor, contribution of carboxylic compounds to the binding of U(VI) on the cell surface or within the cells. As expected, the main peak positions of the spectra of U(VI) bioassociated to *B. napus* cells are similar to those of U(VI) bioassociated with lupine roots from soil culture (Günther et al. 2003), green algae *Chlorella vulgaris* (Günther et al. 2008), and fungi *Schizophyllum commune* 12-43 (Günther et al. 2014) (cf. Tab. SI2). Important for the current study, the authors of each of these prior spectroscopic investigations confirmed the predominant binding of U(VI) to inorganic and/or organic phosphate groups of the biomass. In addition, our TRLFS findings are supported by the EXAFS results of Laurette et al. (2012b), who suggested the complexation of U with intracellular inorganic and organic phosphate residues in *B. napus* and sunflower roots.

**Fig. 7:**
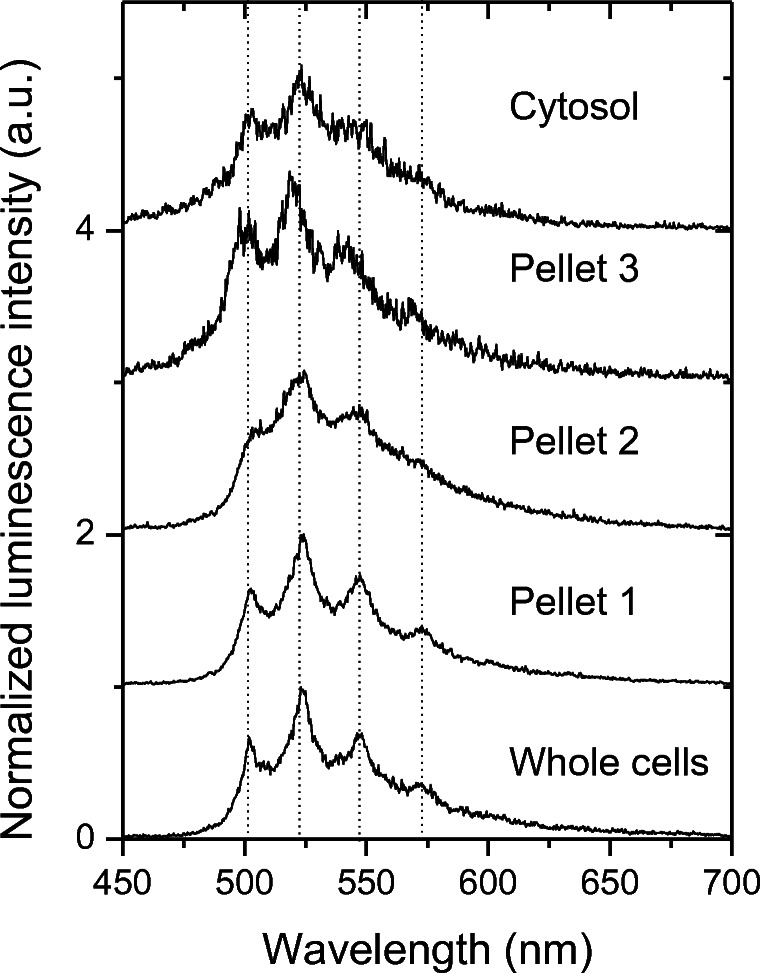
Normalized luminescence spectra of *B. napus* cell fractions of cells that were cultivated in the presence of 200 μM U(VI)

Figure 7 shows the normalized TRLFS spectra of cell fractions suspended in 0.154 M NaNO_3_, which were obtained by fractionation of cells cultivated in the presence of 200 μM U(VI). In agreement with our ICP-MS results (cf. Fig. 3b), U(VI) was detected in all cell fractions, thereby indicating the extra- and/or intracellular bioassociation of U(VI). Note that the spectra of all fractions are similar in their main peak positions and intensity ratios, indicating a similar binding of U(VI). Differences in the signal-to-noise ratios can be attributed to the varying uranium content in the fractions, as well as the different sample amounts available for measurement. Note also that the spectra of the individual cell fractions are comparable to those of the whole cells. Therefore, the similarity of the spectrum for the whole cells when compared with that of Pellet 1, as well as the fact that most of the U(VI) was found in Pellet 1 (Fig. 3), confirms the dominant binding of U(VI) to heavy cell compartments (e.g., the cell wall), which represents an effective protective mechanism of the metabolically active cell compartments against the absorbance of potentially toxic metals. This result agrees with the findings of Laurette et al. (2012a), who described the predominant association of U with the insoluble structures of sunflower plants, e.g., cell walls.

In a related study, El Hayek et al. (2018) employed scanning transmission electron microscopy/energy-dispersive X-ray spectrometry to confirm the predominant binding of uranium in the cell walls of *Brassica juncea*. Nevertheless, it must be noted that for our investigation, the detection of U(VI) in the cytosol fraction indicates that a certain amount of U(VI) was taken up by the cells. This observation is in agreement with Geipel and Viehweger (2015), who studied the speciation of uranium in compartments of living *B. napus* cells in suspension cell cultures. Since different protocols for cell fractionation were used, however, the spectra reported by Geipel and Viehweger are not directly comparable to the spectra in the present work. While the positions of the main emission peaks are similar, the intensity ratios of the peaks differ slightly.

## Conclusions

The results of *B. napus* callus cells grown *in vitro* under Eu(III) or U(VI) stress confirm that *B. napus* cells have a strong capacity to bioassociate both PTMs under the given experimental conditions. Most of the Eu(III) and U(VI) was bound on the cell wall fraction, which could represent the principal mechanism for Eu(III)/U(VI) enrichment. More likely, however, this finding points to the effective protective mechanisms of metabolically active cells against the threat of potentially toxic metals. We also note that, especially under high Eu(III)/U(VI) stress, both metals were found in the cytosol fraction, which does indicate the uptake of Eu(III)/U(VI) into the cells. High Eu(III)/U(VI) stress also showed the slight tendency that the homeostasis of Ca(II) in *B. napus* callus is affected. Moreover, this study confirmed that cell growth was reduced in combination with a decrease in cell vitality. The total cellular phenolic content, which could have increased due to PTM stress, was similar for cells that were exposed to 30 or 200 μM Eu(III), as well as for our controls. Only a slight tendency for a slightly higher phenolic content was found for cells grown in the presence of 20 μM U(VI), whereas at 200 μM U(VI), this value was lower to those of the controls.

Despite the low intensity of the symmetry-forbidden ^7^F_0_ peak in the luminescence emission spectra of Eu(III) bound to *B. napus* cells, the appearance points to the formation of Eu(III) complexes. The occurrence of a bi-exponential luminescence decay confirmed the existence of two Eu(III) coordination environments. The strong intensity of the ^7^F_2_ peak as a measure of changes in the Eu(III) speciation, coupled with the resulting high intensity ratio R_E/M_, indicates the formation of strong Eu(III) complexes. Further analysis of the Eu(III) coordination environment revealed strong inner-sphere Eu(III) species, possibly with organic phosphate and carboxyl groups provided by the *B. napus* cells. In conclusion, *B. napus* cells provide multiple-binding environments for Eu^3+^. Due to the bioassociation of U(VI) onto *B. napus* cells, we observed a significant bathochromic shift of the U(VI) emission bands of the plant cell species compared to the spectra of the reference solution (U(VI) in 0.154 M NaNO_3_). Further, by comparing our findings with model compounds and other biological systems, there is a clear argument to be made for the predominant binding of U(VI) by organic and/or inorganic phosphate groups of the plant biomass. Our TRLFS-based speciation analysis confirmed this biochemical assessment in terms of the predominant binding of both Eu(III) and U(VI) on heavy cell compartments, such as the cell wall. In conclusion, *B. napus* cells are able to accumulate and tolerate potentially toxic metals like Eu(III) and U(VI). As a result, these metals have the potential to enter the food chain and may become a severe health risk for humans. We hypothesize that the PTM tolerance of these cells is likely due to several mechanisms—but most notably the strong binding of the metals within the cell walls that protects the cell compartments against heavy metal toxicity. The results of this study were obtained through combination of biological, biochemical, and spectroscopic methods. We hope that this integrative approach will contribute to an enhanced understanding of the interaction processes between actinides/lanthanides and plants at the molecular level, which is important for modeling the transfer of these elements in the environment.

## Electronic supplementary material

ESM 1 (DOCX 1.18 mb)
