# Supplementary material for: Plant cell (Brassica napus) response to europium(III) and uranium(VI) exposure
Source: Environ Sci Pollut Res Int. 2020 Jun 6;27(25):32048–61. doi: 10.1007/s11356-020-09525-2 (PMC7392935; doi:10.1007/s11356-020-09525-2)
Supplement: Supplementary file 1 — (DOCX 1.18 mb) [file 11356_2020_9525_MOESM1_ESM.docx]

Plant cell (*Brassica napus*) response to europium(III) and uranium(VI) exposure

**Henry Moll^[[1]](#footnote-2)^, Susanne Sachs, and Gerhard Geipel**

*Helmholtz-Zentrum Dresden-Rossendorf, Institute of Resource Ecology, Bautzner Landstraße 400, 01328 Dresden, Germany*

**Supporting Information**

**Table SI1:** Composition of the solid modified Linsmaier and Skoog medium with a reduced phosphate concentration (medium R_red_; ­pH 5.8; adapted from Linsmaier&Skoog 1965).

| **Substance** | **Concentration (mg/L)** | **Substance** | **Concentration (mg/L)** |
| --- | --- | --- | --- |
| NH_4_NO_3_ | 1650 | CuSO_4_×5 H_2_O | 0.005 |
| KNO_3_ | 1900 | CoCl_2_×6 H_2_O | 0.005 |
| MgSO_4_×7 H_2_O | 370 | Nicotinic acid | 0.2 |
| KH_2_PO_4_ | 0.85 | Thiamin×HCl | 2 |
| CaCl_2_ | 332.02 | Pyridoxal×HCl | 0.2 |
| KCl | 9.3 | myo-Inositol | 20 |
| FeNaEDTA | 36.7 | 2,4-Dichlorophenoxyacetic acid | 2 |
| H_3_BO_3_ | 6.2 | 2,4,5-Trichlorophenoxyacetic acid | 2 |
| MnSO_4_×H_2_O | 16.9 | Kinetin | 0.5 |
| ZnSO_4_×7 H_2_O | 8.6 | Sucrose | 30000 |
| KJ | 0.83 | Agar | 15000 |
| Na_2_MoO_4_×2 H_2_O | 0.05 |  |  |

**Table SI2:** Peak maxima of U(VI) bioassociated to *Brassica napus* cells in comparison to selected biological reference systems and U(VI) reference compounds.

|  | **Main emission bands / nm** | | | | | | |
| --- | --- | --- | --- | --- | --- | --- | --- |
| **Whole cells** | | | | | | | |
| Cells grown in the presence of 20 µM U(VI)^a^ | 486.4 ± 0.8 | 500.9 ± 0.3 | | 521.0 ± 0.2 | 545.6 ± 0.3 | 575.0 ± 0.4 | 609.3 ± 0.5 |
| Cells grown in the presence of 200 µM U(VI)^b^ | 485.9 ± 0.8 | 503.2 ± 0.1 | | 523.4 ± 0.1 | 546.3 ± 0.1 | 570.7 ± 0.2 | 598.5 ± 0.4 |
| **Fractions of cells grown in the presence of 200 µM U(VI)** | | | | | | | |
| Pellet 1^c^ | 483.3 ± 0.5 | 502.6 ± 0.1 | | 522.6 ± 0.1 | 545.6 ± 0.1 | 570.5 ± 0.2 | 601.1 ± 0.3 |
| Pellet 2^c^ | 481.4 ± 0.3 | 502.0 ± 0.1 | | 522.2 ± 0.1 | 545.9 ± 0.1 | 569.6 ± 0.2 | 601.0 ± 0.3 |
| Pellet 3^c^ |  | 500.6 ± 0.2 | | 521.3 ± 0.1 | 544.8 ± 0.2 |  |  |
| Cytosol^c^ | 482.8 ± 0.3 | 500.9 ± 0.1 | | 522.4 ± 0.1 | 546.6 ± 0.2 | 572.8 ± 0.3 | 607.3 ± 0.5 |
| **U(VI) in 0.154 M NaNO_3_ at pH 5.8** | | | | | | | |
| 20 µM U(VI) | 474.3±1.5 | 496.2±0.1 | | 514.2±0.1 | 534.0±0.1 | 557.8±0.2 | 586.3±0.7 |
| 200 µM U(VI) | 478.3±1.6 | 497.7±0.1 | | 514.3±0.1 | 533.1±0.1 | 556.8±0.2 | 585.2±0.5 |
| **Selected biological reference systems** | | | | | | | |
| U(VI) in lupine roots from soil culture (Günther et al. 2003) | 489.2 | 502.6 | | 525.0 | 547.6 | 575.6 |  |
| U(VI) associated to *Chlorella vulgaris* at pH 6 (Günther et al. 2008) | 488.1 | 504.0 | | 524.9 | 547.5 | 571.7 | 597.4 |
| U(VI) associated to *Schizophyllum commune* 12-43 at pH 6 (Günther et al. 2014) | 487.3 | 500.2 | | 521.6 | 546.2 | 575.5 |  |
| U(VI) associated to a fraction of heavy cell components of *B. napus* cells including plastids and mitochondria (Geipel&Viehweger 2015) | 481.6 | 498.1 | | 518.9 | 541.0 | 564.1 |  |
| U(VI) in a cytoplasm fraction of *B. napus* cells measured at room temperature (Geipel&Viehweger 2015) | 481.2 | 498.7 | | 520.2 | 543.8 |  |  |
| **Selected U(VI) reference compounds** | | | | | | | |
| (UO­_2_)_3_(OH)_5_^+^ (Sachs et al. 2007) | 484 | | 498 | 514 | 534 | 557 | 583 |
| UO_2_HPO_4_ (Billard&Geipel 2008) |  | | 497 | 519 | 543 | 570 |  |
| (UO_2_)_x_(PO_4_)_y_ (Billard&Geipel 2008) | 488 | | 503 | 524 | 547 | 573 | 601 |
| UO_2_-o-phospho-L-threonine (Günther et al. 2006) | 483.7 | | 501.8 | 523.4 | 546.8 | 572.6 | 601.0 |
| UO_2_-fructose 6-phosphate (Koban et al. 2004) | 478.9 ± 0.4 | | 497.1 ± 0.3 | 519.0 ± 0.3 | 543.3 ± 0.3 | 568.9 ± 0.9 | 598 ± 2 |
| UO_2_(glycine)_2_^2+^ (Günther et al. 2007) | 478.7 | | 495.3 | 516.7 | 540.6 | 565.0 | 594.4 |
| UO_2_(malonate) (Brachmann et al. 2002) | 477 | | 494 | 515 | 540 | 564 | 594 |
| UO_2_(malonate)_2_^2-^ (Brachmann et al. 2002) | 479 | | 496 | 517 | 542 | 566 | 597 |

^a^ Fit of an individual sample spectrum.

^b^ Fit of an average spectrum of five individual samples.

^c^ Fit of an average spectrum of two individual samples.





**Fig. SI1.** Total phenolic content of *B. napus* callus cells after 6 weeks of exposure to Eu(III) or U(VI) in comparison to control samples. Data represent mean values ± SD of 25 individual samples for control and each heavy metal concentration from 8 independent experiments. The cross indicates a tendency (p-value: 0.22).


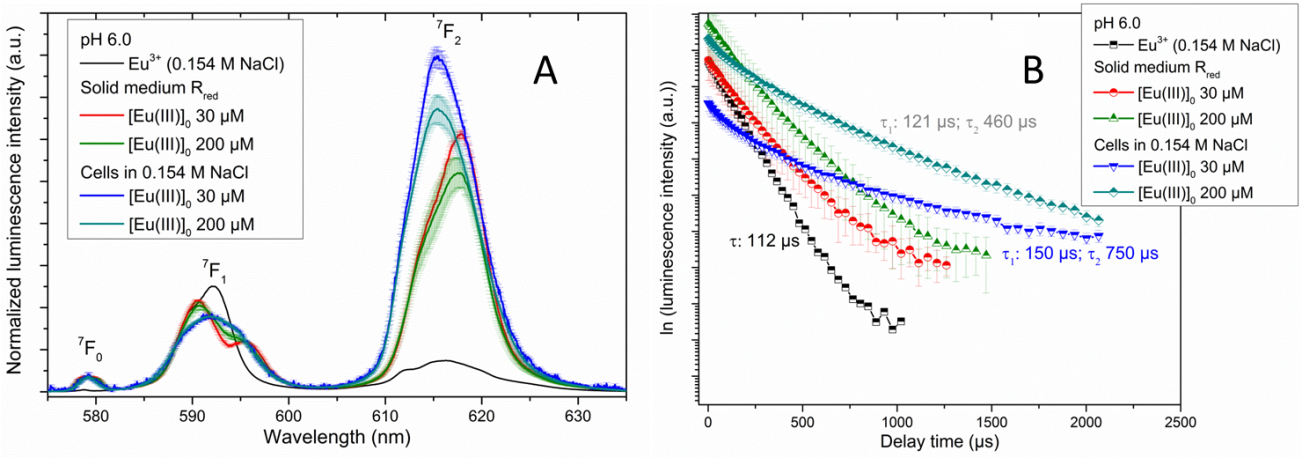


**Fig. SI2.** A) Average luminescence emission spectra and B) time dependence of the luminescence decay of Eu(III) in the *B. napus* callus cell system after 6 weeks of incubation.

**References**

Billard I, Geipel G (2008) Luminescence analysis of actinides: Instrumentation, applications, quantification, future trends, and quality assurance. Springer Ser Fluoresc 5:465-492

Brachmann A, Geipel G, Bernhard G, Nitsche H (2002) Study of uranyl(VI) malonate complexation by time resolved laser-induced fluorescence spectroscopy. Radiochim Acta 90:147-153

Geipel G, Viehweger K (2015) Speciation of uranium in compartments of living cells. Biometals 28:529-539

Günther A, Bernhard G, Geipel G, Reich T, Roßberg A, Nitsche H (2003) Uranium speciation in plants. Radiochim Acta 91:319-328

Günther A, Geipel, G, Bernhard G (2006) Complex formation of U(VI) with the amino acid *L*-threonine and the corresponding phosphate ester O-phospho-*L*-threonine. Radiochim Acta 94:845-851

Günther A, Geipel G, Bernhard G (2007) Complex formation of uranium(VI) with the amino acids L-glycine and L-cysteine: A fluorescence emission and UV-VIS absorption study. Polyhedron 26:59-65

Günther A, Raff J, Geipel G, Bernhard G (2008) Spectroscopic investigations of U(VI) species sorbed by the green algae *Chlorella vulgaris*. Biometals 21:333-341

Günther A, Raff J, Merroun ML, Roßberg A, Kothe E, Bernhard G (2014) Interaction of U(VI) with *Schizophyllum commune* studied by microscopic and spectroscopic methods. Biometals 27:775-785

Koban A, Geipel G, Roßberg A, Bernhard G (2004) Uranium(VI) complexes with sugar phosphates in aqueous solution. Radiochim Acta 92:903-908

Sachs S, Brendler V, Geipel G (2007) Uranium(VI) complexation by humic acid under neutral pH conditions studied by laser-induced fluorescence spectroscopy. Radiochim Acta 95:103-110

Linsmaier EM, Skoog F (1965) Organic growth factor requirements of tobacco tissue cultures. Physiol Plant 18:100-127

1. Address correspondence to Henry Moll, Institute of Resource Ecology, Helmholtz-Zentrum Dresden-Rossendorf, Bautzner Landstrasse 400, 01328 Dresden, Germany. E-mail: [h.moll@hzdr.de](mailto:h.moll@hzdr.de). [↑](#footnote-ref-2)
